# Discovery of interspecific alloparental care of silver‐eared mesia nestlings by a mountain bulbul

**DOI:** 10.1002/ece3.11641

**Published:** 2024-07-02

**Authors:** Jianping Liu, Bin Li, Wei Liang

**Affiliations:** ^1^ College of Biological Sciences and Engineering, North Minzu University Yinchuan China; ^2^ Ministry of Education Key Laboratory for Ecology of Tropical Islands, Key Laboratory of Tropical Animal and Plant Ecology of Hainan Province College of Life Sciences, Hainan Normal University Haikou China

**Keywords:** interspecific alloparental care, mountain bulbul, nest sanitation behavior, silver‐eared mesia

## Abstract

Although brood parasitism has been well documented among bird species, interspecific alloparenting, which is parenting behavior of adult individuals of one species toward the progeny of another species, is increasingly being reported. However, compared with the many reports of interspecific alloparenting behavior in North America and Europe, this phenomenon is less well known in China, with only two prior cases of interspecific alloparenting behavior in birds having been recorded. On June 23, 2022, we observed an instance of interspecific alloparental care provided by a mountain bulbul (*Ixos mcclellandii*) towards silver‐eared mesia (*Leiothrix argentauris*) nestlings in Caihu Village, Jingdong County, Yunnan Province, southwestern China. We recorded 19.5 h of footage during the period in which the mountain bulbul provided care for the nestlings with the aim of documenting detailed observations of interspecific alloparenting to contribute to our overall understanding of this behavior. The alloparenting behavior of the mountain bulbul lasted for at least 5 days. During this period, both silver‐eared mesia parents fed their nestlings 157 times and removed their nestlings' fecal sacs 5 times, while the mountain bulbul fed the nestlings 30 times and removed the nestlings' fecal sacs 4 times. In addition, the male silver‐eared mesia parent chased the mountain bulbul away during nestling feeding. As there was no life history information for the mountain bulbul at that time, we were unable to directly determine why it exhibited interspecific alloparental care. Regardless of the reason for the mountain bulbul's behavior, these findings provide valuable information for future studies on the reproductive ecology of these two bird species.

## Graphical abstract

Parenting behavior by adult individuals of one species for the progeny of another species is widespread but less well known in China. We observed a mountain bulbul (*Ixos mcclellandii*) helping the silver‐eared mesia (*Leiothrix argentauris*) care for nestlings. The alloparenting behavior of the mountain bulbul lasted for at least 4 days and the male silver‐eared mesia parent chased away the mountain bulbul during nestling feeding.

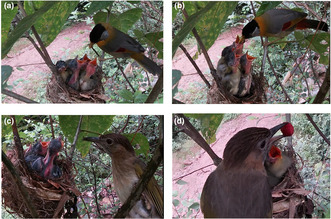

## 1 INTRODUCTION

Interspecific alloparental care is uncommon in birds. However, there have been increasing reports of interspecific alloparental care on a global scale (Halley & Heckscher, 2013; Jiang et al., 2016; Levey & del Coro Arizmendi, 2021; Luo et al., 2018; Schaeffer et al., 2009). Apart from interspecific brood parasitism, only 186 examples of interspecific alloparental care behavior have been reported, involving 107 species from 41 families in 10 orders, which represents less than 2% of the total number of birds worldwide (Harmáčková, 2021). Furthermore, prior to the current study, only two cases of interspecific alloparental care had been reported in China (Jiang et al., 2016; Luo et al., 2018). Detailed records of interspecific alloparental care can contribute to our overall understanding of the evolution of this behavior, including whether it is limited to a few bird species or prevalent across avian species and whether it is restricted to a particular region or exhibited worldwide. Furthermore, as it is unclear why individual birds engage in parental care at nests that are not their own, investigating the taxonomic distribution and habitat associations of alloparenting may elucidate the underlying reasons for this behavior. Therefore, in this study, we aimed to provide detailed observations of a mountain bulbul (*Ixos mcclellandii*) that engaged in interspecific alloparental care of silver‐eared mesia (*Leiothrix argentauris*) nestlings in southwestern China.

## 2 METHODS

The study site was located in Caihu Village (24°17′37″ N, 100°35′22″ E), Jingdong County, Yunnan Province, southwestern China. The village has an elevation of 1767 m, the annual mean temperature is 15°C, and the annual precipitation is 1500 mm. The study site is located in the deep mountains of a sparsely populated area and is characterized mainly by natural secondary and artificial forests.

On June 15, 2022, we discovered a silver‐eared mesia nest in Caihu Village. The silver‐eared mesia nestlings were hatching, with two hatched nestlings and two eggs in the nest. To investigate the feeding behavior of silver‐eared mesias, we used a GoPro HERO 9 action camera (GoPro, San Mateo, CA, USA) combined with a 10,000 mAh external power supply (TP‐D094, Pisen, Guangdong Pinsheng Electronics Co., Ltd., China) to record the silver‐eared mesia nest. The recording equipment was tied to a tree 1.5 m from the nest. The silver‐eared mesia nestlings fledged on June 27, 2022. Therefore, the recording period was from June 15 to 27, 2022. The daily recording duration was not fixed, starting as early as 05:00 and ending as late as 20:00 (GMT + 8). The shortest daily recording duration was 1 h and the longest daily recording duration was 8 h. As male and female silver‐eared mesias differ in coloration (Zhao, 2001), we were able to differentiate between the parents and record their feeding events separately. Through the video recordings, we observed a mountain bulbul providing interspecific alloparental care to the silver‐eared mesia nestlings. Mountain bulbuls typically build their nests on the lateral branches of tall trees or on shrubs and small trees in the understory, with nest heights ranging from 1.2 to 12 m above the ground level (Zhao, 2001). Bulbul nests are cup‐shaped and primarily made of grass stems, leaves, and roots (Zhao, 2001). Despite a systematic search for mountain bulbul nests, we were unable to locate any active nests in the study area. Due to the similar appearance of male and female mountain bulbuls, we were unable to identify the sex of the individual that provided interspecific alloparental care to the mesia nestlings.

We imported the videos recorded using the GoPro into a computer (Thinkpad X1 Carbon, Lenovo, China) and used video playback to document the behaviors of the silver‐eared mesias and mountain bulbul at the nest. We defined feeding behavior as an adult bird carrying food and delivering it to the mouths of the chicks, whereas nest‐cleaning behavior was defined as an adult bird removing fecal sacs left by the chicks after feeding.

## 3 RESULTS

The recordings showed that the mountain bulbul first provided interspecific alloparental care to the silver‐eared mesia nestlings on June 23. At this time, the four nestlings were 8 days old. As the recordings did not cover the entire day, it was unclear whether the mountain bulbul provided care to the nestlings before this date. However, from June 23 to 27, the recordings showed that the mountain bulbul assisted in feeding the nestlings every day. Therefore, the mountain bulbul engaged in feeding behavior for at least 5 days, until the nestlings fledged on June 27.

**FIGURE 1:**
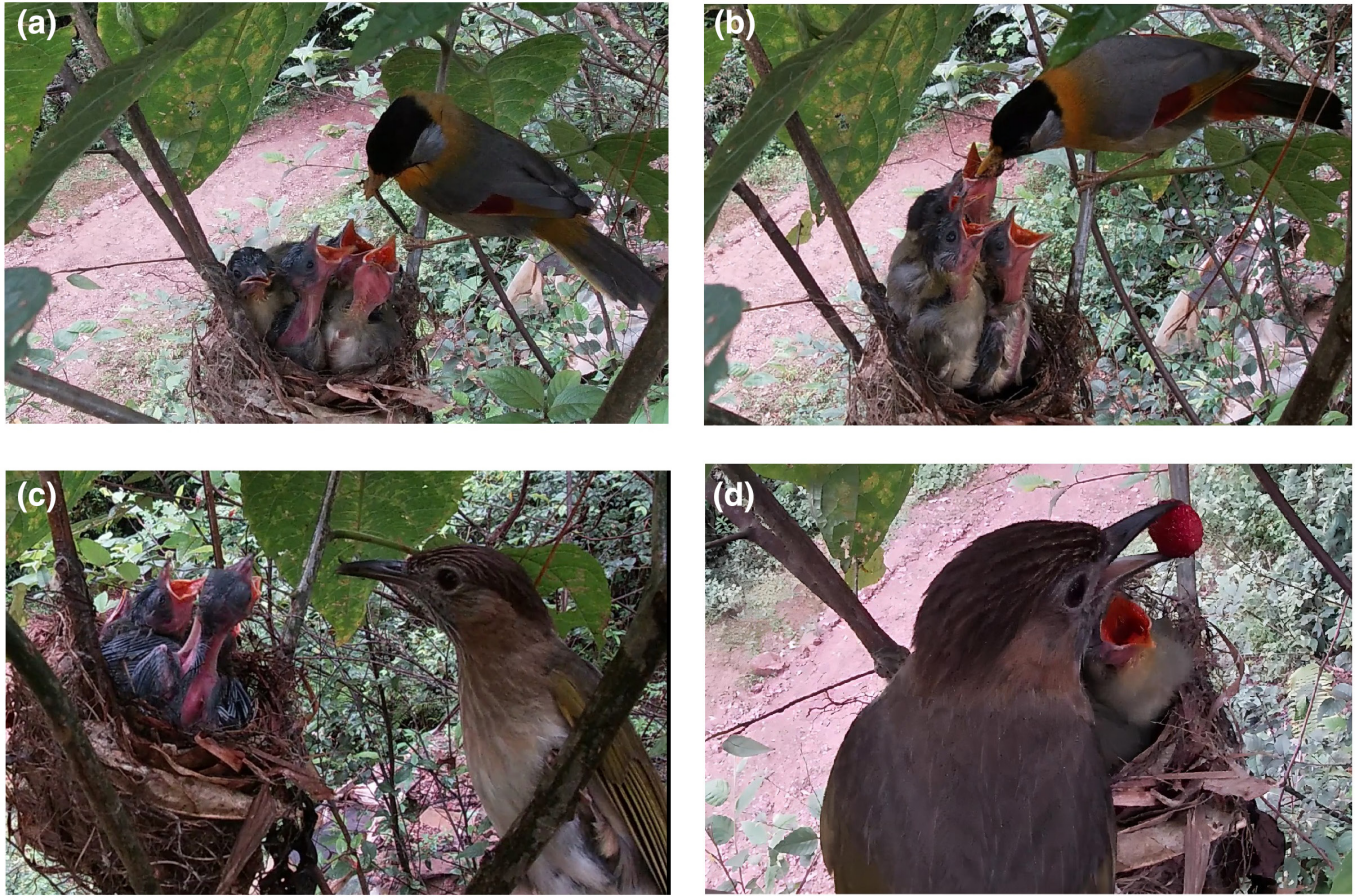
Silver‐eared mesia (*Leiothrix argentauris*) nestlings fed by both parents and an alloparent: (a) female and (b) male silver‐eared mesia feeding moths to their nestlings; (c) first documented visit by the alloparent, a mountain bulbul (*Ixos mcclellandii*), to the nest; and (d) a mountain bulbul feeding the silver‐eared mesia nestlings.

**FIGURE 2:**
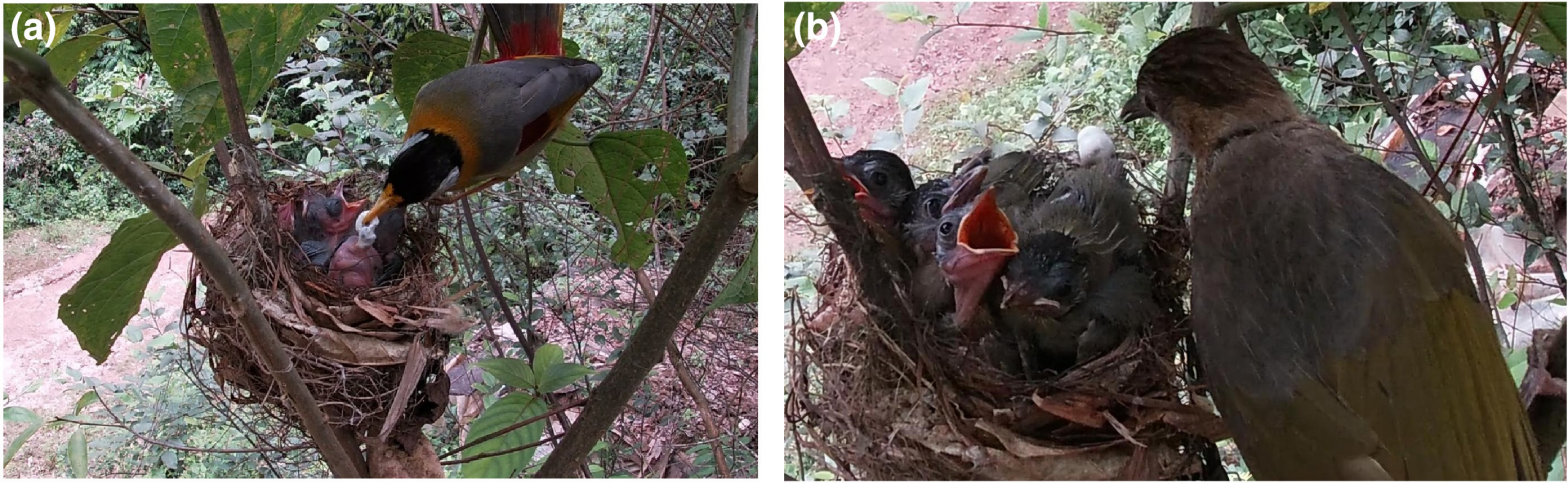
(a) Silver‐eared mesias and (b) the alloparent mountain bulbul removing the fecal sacs after feeding the silver‐eared mesia nestlings.

We recorded 48.3 h during the 13‐day nestling period of the silver‐eared mesia. During this time, the female and male silver‐eared mesias fed their nestlings 133 and 106 times, respectively (Video S1). We recorded 19.5 h during the 5 days that the mountain bulbul provided interspecific alloparental care. During this period, the female silver‐eared mesia fed the nestlings 88 times, the male silver‐eared mesia fed the nestlings 69 times, and the mountain bulbul fed the nestlings 30 times (Video S2). The food fed to the nestlings by the silver‐eared mesia parents mainly consisted of Lepidoptera, Odonata, and Orthoptera adults and larvae (Figure 1). In addition to feeding the silver‐eared mesia nestlings insects, the mountain bulbul fed them red and black berries (Figure 2). The male and female silver‐eared mesia parents exhibited nest‐sanitation behavior and removed nestling fecal sacs after feeding. The mountain bulbul also provided interspecific alloparental care to the silver‐eared mesia nestlings by removing their fecal sacs. The mountain bulbul removed fecal sacs from the nest four times during the observation period (Figure 2).

In addition, the male silver‐eared mesia was observed chasing the mountain bulbul away from the nest (Video S3). After feeding the nestlings, the mountain bulbul typically remained in the area around the nest, and when it observed that the male parent was near the nest, it immediately flew away. In one recorded interaction, the mountain bulbul did not fly away after feeding the nestlings and was attacked by the male silver‐eared mesia. We did not observe the female silver‐eared mesia rejecting the presence of the mountain bulbul.

## 4 DISCUSSION

In birds, interspecific parental care is often observed in the form of brood parasitism, wherein a bird lays its eggs in another bird's nest to induce it to raise the chicks as its own. However, feeding the offspring of other birds can also occur in other situations (Jiang et al., 2016; Konter, 2012; Levey & del Coro Arizmendi, 2021; Luo et al., 2018; Mann, 2017). For example, there are many records of interspecific alloparental care in songbirds (Fiss et al., 2016; Halley & Heckscher, 2013; Harmáčková, 2021; Krištín, 2009). In this study, we observed a mountain bulbul feeding silver‐eared mesia nestlings for a period of 5 days beginning on June 23, 2022. Attempts to find a mountain bulbul nest near the silver‐eared mesia nest were unsuccessful. Therefore, owing to limited knowledge of the mountain bulbul's life history, the underlying factors driving interspecific alloparental care behaviors in mountain bulbuls remain uncertain. However, video evidence showed that the mountain bulbul fed the silver‐eared mesia nestlings less frequently than their parents did. Additionally, there were differences between the male and female silver‐eared mesia parents in their tolerance of the presence of the mountain bulbul near their nest. The male silver‐eared mesia parent was observed attacking the mountain bulbul when it remained near the nest after feeding the nestlings, whereas the female parent did not exhibit this behavior.

Regarding interspecific alloparental care, Shy (1982) listed eight possible reasons for this behavior: (1) the nest contained the progeny of two species (i.e., a mixed nest), which may occur when different species lay eggs in the same nest due to limitations in nesting sites (Barrientos et al., 2015); (2) the bird was physiologically in a parenting state but did not have a nest (e.g., nest was predated), which resulted in the transfer of parental care behaviors to a nearby nest (Haucke, 2015; Riedman, 1982; Skutch, 1999); (3) birds tend to be attracted by neighboring nests, and the nests of individuals who provided interspecific alloparental care were in close proximity to the nest of other birds (Jiang et al., 2016); (4) the individuals were stimulated by the loud begging calls of the nestlings from another species; (5) the progeny of one species were orphaned and adopted by individuals of another species; (6) the mate of a male bird was incubating eggs, so the male fed the nestlings of other birds; (7) the individuals were either unmated or did not have offspring of their own; and (8) other unspecified reasons. The possibility that the mountain bulbul was attracted to a neighboring silver‐eared mesia nest was not supported by the evidence, as attempts to find a mountain bulbul nest within 100 m of the silver‐eared mesia nest were unsuccessful. At the study site, the nesting preferences of mountain bulbuls and silver‐eared mesias might overlap. It is also possible that the nest of the mountain bulbul was predated upon or destroyed, although we found no evidence to support this. However, as these are common reasons for interspecific alloparental care behavior in other bird species (Haucke, 2015; Heber, 2013), one or more of these factors may explain the mountain bulbul's provision of interspecific alloparental care to the silver‐eared mesia nestlings. In addition, it is possible that the mountain bulbul was stimulated by the vocalizations of the silver‐eared mesia nestlings, compelling it to engage in alloparenting behavior (Shy, 1982). Notably, these reasons are not mutually exclusive, and more than one factor may have influenced the bulbul's behavior. Regardless of why the bulbul exhibited interspecific alloparental care, it was observed responding to the calls of the silver‐eared mesia chicks. In the recordings, the silver‐eared mesia nestlings exhibited begging behavior towards birds near their nest, regardless of whether these birds were their parents or not. Consequently, the mountain bulbul was observed feeding the silver‐eared mesia nestlings 30 times over five consecutive days. This supports the likelihood that the nest of the mountain bulbul was predated on, leading the bulbul to transfer its parenting behavior to nestlings in a nearby nest. A previous study suggested that the desire to feed progeny may be a reason for interspecific alloparenting in mountain bulbuls (Harmáčková, 2021). Similar to the reasons listed by Shy (1982), this may occur in cases where the individual providing interspecific alloparental care is a mateless bird or a mated male bird whose mate is incubating eggs. It is possible that the mountain bulbul may not have had its own progeny to feed and therefore, fed the progeny of the silver‐eared mesias. The mountain bulbul observed in this study showed parental care towards chicks of another species, suggesting that they cannot differentiate between their own chicks and those of different species, which is similar to the behavior of other birds (Peek et al., 1972; Turtumøygard & Slagsvold, 2010; Tyller et al., 2018). This failure to recognize differences in progeny may be one of the reasons for the evolution of interspecific brood parasitism in birds. For example, mountain bulbul nests have been observed to be parasitized by the common cuckoo (*Cuculus canorus*) (Lowther, 2017).

Although we were unable to definitively identify the reasons for the provision of parental care by the mountain bulbul towards the silver‐eared mesia nestlings, we hypothesize that overlap in the breeding time and nest environment of mountain bulbuls and silver‐eared mesias, breeding failure in the mountain bulbul, and stimulation by vocalizations from the silver‐eared mesia nestlings may have been the reasons why the mountain bulbul provided parental care to the silver‐eared mesia nestlings. Regardless of the behavioral mechanisms underlying interspecific alloparenting behavior in mountain bulbuls, these findings provide valuable information for future studies on the reproductive ecology of these two bird species.

## AUTHOR CONTRIBUTIONS

**Jianping Liu:** Data curation (equal); formal analysis (lead); methodology (equal); supervision (equal); writing – original draft (lead). **Bin Li:** Data curation (equal); investigation (lead). **Wei Liang:** Conceptualization (equal); supervision (equal); writing – review and editing (lead).

## FUNDING INFORMATION

This work was supported by the National Natural Science Foundation of China (Nos. 32160242 to JL and 32270526 to WL). Additionally, WL was supported by the specific research fund of The Innovation Platform for Academicians of Hainan Province.

## CONFLICT OF INTEREST STATEMENT

The authors declare that they have no competing interests.

## Supporting information


Video S1.

Video S2.

Video S3.

## Data Availability

The data that supports the findings of this study are provided in Supplementary Material (Videos [Link], [Link]).
